# Diagnostic Concordance and Misclassification of Pre‐Eclampsia in a Malaria‐Endemic Setting: A Comparative Cross‐Sectional Study

**DOI:** 10.1155/bmri/8646264

**Published:** 2026-04-26

**Authors:** Bismark Opoku Mensah, Abena Adjei Serwaa, Millicent Kwour Dugbatey, Ernestina Anim

**Affiliations:** ^1^ Department of Biological Sciences, University of Worcester, Worcester, UK, worcester.ac.uk; ^2^ Department of Population and Reproductive Health, KNUST School of Public Health, Kumasi, Ghana; ^3^ Department of Public Health, Komfo Anokye Teaching Hospital, Kumasi, Ghana, kathhsp.org; ^4^ Centre for Graduate Studies and Research, KAFF University, Accra, Ghana; ^5^ Department of Nursing, All Nations University, Koforidua, Ghana

**Keywords:** diagnostic accuracy, low-resource settings, malaria, maternal health, misclassification, pre-eclampsia, proteinuria

## Abstract

**Background:**

In malaria‐endemic settings, overlapping clinical features between malaria and pre‐eclampsia complicate accurate diagnosis of pre‐eclampsia during routine antenatal care. Misclassification may delay initiation of appropriate management or lead to unnecessary interventions. This study evaluated diagnostic concordance between routine clinical assessment and predefined reference criteria and examined the contribution of malaria to diagnostic discordance.

**Methods:**

A cross‐sectional study was conducted amongst 618 pregnant women clinically diagnosed with malaria, pre‐eclampsia or both across primary, secondary and tertiary health facilities. Routine diagnoses were compared with reference classification based on predefined criteria. Diagnostic concordance was assessed using *χ* ^2^ testing and Cohen′s kappa coefficient. Sensitivity, specificity, positive predictive value (PPV), and negative predictive value (NPV) for the routine detection of pre‐eclampsia were calculated. Multivariable linear and logistic regression analyses were performed to evaluate associations between log‐transformed parasite density and diagnostic misclassification.

**Results:**

Overall concordance between routine and reference classification was 50.2% (*κ* = 0.233, *p* < 0.001). Routine diagnosis of pre‐eclampsia demonstrated a sensitivity of 61.8% and specificity of 62.2%, with a PPV of 65.9% and an NPV of 57.9%. Malaria parasite density was not independently associated with systolic or diastolic blood pressure but was associated with higher ln(uPCR) (*β* = 0.17, *p* < 0.001). Malaria parasite density was not an independent predictor of diagnostic discordance.

**Conclusion:**

Routine diagnosis of pre‐eclampsia in malaria‐endemic settings demonstrates moderate diagnostic performance, with considerable misclassification, particularly in the presence of concurrent malaria.

## 1. Introduction

Pre‐eclampsia (PE) remains a leading cause of maternal and perinatal morbidity and mortality worldwide. It complicates an estimated 2%–8% of pregnancies worldwide [1] and accounts for a substantial proportion of maternal deaths, especially in low‐ and middle‐income countries [2, 3]. Sub‐Saharan Africa bears a disproportionate burden, where delayed recognition, limited diagnostic capacity and restricted access to timely intervention exacerbate adverse maternal and neonatal outcomes [4, 5]. Early and accurate identification of PE during routine antenatal care (ANC) is therefore essential to prevent progression to severe complications.

In malaria‐endemic regions, clinical evaluation of PE is complicated by the high prevalence of malaria infection [6]. Malaria and PE share overlapping clinical and pathophysiological features, including endothelial dysfunction, systemic inflammation and, in some cases, renal involvement manifested as proteinuria [7]. This biological and clinical overlap increases the potential for diagnostic ambiguity, particularly in resource‐constrained settings where assessment may depend on symptom evaluation, single blood pressure measurements and qualitative dipstick proteinuria testing [8, 9].

Despite these challenges, there is limited empirical evidence quantifying the degree of diagnostic concordance between routine assessment and more rigorous reference diagnostic criteria in malaria‐endemic populations. It also remains uncertain whether malaria parasite burden directly contributes to elevations in blood pressure or proteinuria sufficient to drive diagnostic discordance or whether misclassification primarily reflects contextual diagnostic limitations.

This study sought to address these gaps. The primary objective was to evaluate diagnostic concordance between routine clinical diagnosis and predefined reference classification for PE across multiple levels of care. The secondary objectives were to assess the diagnostic performance of routine PE detection and to examine the contribution of malaria parasite density to blood pressure elevation, proteinuria and diagnostic discordance.

Unlike prior studies that have largely focused on the pathophysiological overlap or biomarker‐based differentiation between malaria and PE, this study examines diagnostic concordance and the performance of routine PE detection in malaria‐endemic settings, with particular attention to misclassification in the presence of coinfection.

## 2. Materials and Methods

### 2.1. Study Design and Setting

This comparative cross‐sectional study was conducted in Ghana from September 2023 to July 2024. The study was conducted at Community‐based Health Planning and Services (CHPS) compounds and district hospitals, which are the primary points of care for pregnant women in rural and periurban areas. Regional hospitals that serve as referral centres for obstetric and medical complications were also included. The study was designed to reflect routine ANC and referral practices in Ghana, where most pregnant women first visit CHPS compounds or district hospitals and, if needed, are referred to higher level facilities for the management of complex conditions such as PE and severe malaria.

### 2.2. Ethical Considerations

Ethical approval was obtained from the Committee on Human Research Publications and Ethics of Kwame Nkrumah University of Science and Technology (CHRPE/AP/677/23). Written informed consent was obtained from all participants. Where severe hypertension or other life‐threatening features were identified during reference assessment, findings were immediately communicated to the attending clinical team in accordance with predefined safety escalation procedures.

### 2.3. Sample Size Determination

The sample size was determined to estimate diagnostic discordance between routine clinical diagnosis and the study reference standard. With no prior data on estimates of diagnostic discordance in this population, a conservative proportion of *p* = 0.50 was used to maximise variance and provide the largest required sample size. The minimum sample size was calculated using the following:

n=Z2p1−pd2

At a 95% confidence level and absolute precision of ±4%, the minimum required sample size was 600. To account for incomplete data, specimen loss or laboratory assay failures, the sample size was inflated by 10%, resulting in a target enrolment of 660 participants.

### 2.4. Study Population and Participant Recruitment

#### 2.4.1. Frontline Enrolment

Consecutive pregnant women presenting at participating CHPS compounds or district hospitals were screened for eligibility if they were at ≥ 20 weeks′ gestation and presented with suspected malaria, symptoms suggestive of hypertensive disorders of pregnancy or both. These symptoms included fever or history of fever, headache, malaise, chills, oedema, visual disturbance, epigastric or right upper quadrant pain, dyspnoea, reduced foetal movements or elevated blood pressure at presentation.

#### 2.4.2. Referral‐Level Enrolment

In parallel, consecutive pregnant women arriving at participating regional hospitals with a referral for diagnosis of PE or malaria in pregnancy with hypertensive features were also enrolled.

### 2.5. Eligibility and Inclusion Criteria

Eligible participants were pregnant women aged ≥ 18 years with a gestational age between 20 and 42 weeks. Gestational age eligibility was restricted to participants with ultrasound‐confirmed dating, obtained either from antenatal records documenting prior early obstetric ultrasound or confirmed by the research team where applicable. Symptomatic participants were eligible if they had been clinically diagnosed by attending healthcare providers to have malaria, PE or both conditions based on routine clinical assessment in accordance with standard practice at participating CHPS compounds, district hospitals or regional hospitals.

### 2.6. Exclusion Criteria

Participants requiring immediate life‐saving intervention at presentation were excluded from the study. Additional exclusion criteria included documented or suspected chronic kidney disease, systemic infections other than malaria, HIV infection and urinary tract infection at enrolment or within the preceding 2 weeks. Participants who had received or were receiving antimalarial drugs, antibiotics, antiretroviral therapy, antihypertensive medications or anti‐inflammatory drugs at the time of enrolment were also excluded.

### 2.7. Clinical Assessment

#### 2.7.1. Routine (Index) Diagnosis

At first contact, the initial working diagnosis and management decisions made by attending healthcare providers were recorded before any study‐directed investigations were performed. This included documentation of whether the participant was managed as having malaria, PE or both conditions. The basis for the routine clinical diagnosis was clinical judgement, malaria rapid diagnostic testing (RDT) and/or microscopy, blood pressure measurement and urine dipstick testing, as performed in accordance with the facility′s practice.

#### 2.7.2. Reference Assessment and Data Collection

All enrolled participants underwent a standardised reference assessment conducted by trained healthcare professionals and the research team. This reference assessment was performed independently of routine clinical diagnoses. Research staff conducting the assessment were blinded to the routine clinical (index) diagnosis recorded by the attending healthcare providers.

Sociodemographic and obstetric information, such as maternal age, parity and gestational age, was collected using a structured case report form. Gestational age was confirmed by early ultrasound dating.

Blood pressure was measured using a validated automated sphygmomanometer (Omron HEM‐7155‐E, Omron Healthcare, Japan). Measurements were taken with participants seated, back supported, feet flat on the floor and arm supported at heart level after a minimum rest period of 5 min. Two blood pressure readings were obtained at 1–2 min intervals. The mean of the final two readings was used for analysis. Devices were calibrated periodically according to manufacturer recommendations.

Maternal weight and height were measured using a calibrated Seca 813 digital scale and Seca 213 stadiometer (Seca GmbH, Hamburg, Germany), respectively. Proteinuria was assessed using spot urine protein‐to‐creatinine ratio (uPCR). Malaria infection was assessed using malaria RDTs and blood film microscopy.

### 2.8. Case Definitions

PE was defined in accordance with the International Society for the Study of Hypertension in Pregnancy (ISSHP) guidelines as new‐onset hypertension after 20 weeks′ gestation (systolic blood pressure [SBP] ≥ 140 mmHg and/or diastolic blood pressure [DBP] ≥ 90 mmHg) accompanied by proteinuria, defined as a spot uPCR ≥ 0.3 mg/mg, or clinical features consistent with maternal end‐organ involvement. Confirmed malaria was defined as a positive malaria RDT with parasite detection on thick and thin blood film microscopy.

Based on the reference assessment, participants were classified into four mutually exclusive diagnostic groups: PE without malaria, PE with malaria coinfection, malaria without PE and normotensive, malaria‐negative.

### 2.9. Urine Sample Collection and Processing

A spot midstream urine sample was collected from each participant between 9:00 and 11:00 AM into a sterile container. Samples were transported to the laboratory immediately under cold‐chain conditions. They were centrifuged at 1000 × g for 5 min, and the supernatant was aliquoted for subsequent analysis.

### 2.10. Laboratory Analyses

#### 2.10.1. Quantification of Urine Protein and Creatinine

Urine supernatants were analysed immediately or within 24 h of collection following refrigerated storage at 2°C–8°C. Urinary protein and creatinine concentrations were quantified from the same centrifuged urine supernatant using the Mindray BA‐88A semiautomated clinical chemistry analyser (Mindray Bio‐Medical Electronics Co., Shenzhen, China). Urine protein was measured using a colourimetric pyrogallol red–molybdate assay, whilst urine creatinine was determined using an enzymatic creatinine assay, in accordance with the manufacturers′ instructions.

The uPCR was calculated by dividing the urinary protein concentration by the urinary creatinine concentration and expressed as milligrammes per milligramme. A uPCR value of **≥** 0.3 mg/mg was used to define significant proteinuria.

#### 2.10.2. Malaria Diagnosis

At enrolment, malaria RDT was performed using CareStart Malaria HRP2/pLDH Combo RDT (Access Bio Inc., United States) in accordance with the manufacturer′s instructions and routine national malaria control programme guidelines. In addition, thick and thin blood film microscopy was performed. Thick blood films were used for parasite detection and quantification, whilst thin films were used for parasite species identification. Although microscopy allows for the identification of *Plasmodium* species, malaria was not stratified by subtype in this study. This approach was adopted to reflect routine clinical practice, where species differentiation is not typically used to guide initial diagnostic classification. Moreover, given the predominance of *Plasmodium falciparum* in the study setting, subtype classification was unlikely to substantially influence diagnostic interpretation.

Peripheral blood smears were prepared using standard techniques, air‐dried and stained with Giemsa stain. Slides were examined by trained laboratory personnel who were blinded to the routine clinical diagnosis. Parasite density was estimated by counting the number of parasites per 200 leukocytes on thick film and expressed as parasites per microlitre of blood, assuming a standard leukocyte count of 8000/*μ*L.

### 2.11. Statistical Analysis

All statistical analyses were conducted using jamovi (Version 2.7.12) and IBM SPSS Statistics (Version 22). SPSS was used for data management, descriptive statistics and regression analyses, whilst jamovi was used to support data exploration and verification of statistical outputs. A two‐sided *p* value < 0.05 was considered statistically significant. Continuous variables were assessed for normality using histograms and the Shapiro–Wilk test.

Baseline characteristics and clinical parameters were compared using the Kruskal–Wallis test for continuous variables and *χ* ^2^ tests for categorical variables. Parasite density comparisons between malaria‐positive groups were performed using the Mann–Whitney *U* test.

Diagnostic concordance between routine and reference classifications was assessed using contingency tables, Pearson′s *χ* ^2^ test and Cohen′s kappa coefficient. Sensitivity, specificity, positive predictive value (PPV), and negative predictive value (NPV) were derived from 2 × 2 tables.

Multivariable linear regression was used to assess associations between log10‐transformed parasite density and SBP, DBP and natural log‐transformed uPCR (ln[uPCR]), adjusting for maternal age, gestational age, body mass index (BMI) and reference diagnosis. Predictors of diagnostic discordance were examined using multivariable logistic regression with misclassification (routine vs. reference disagreement) as the binary outcome. Adjusted odds ratios (aORs) with 95% confidence intervals (CIs) were reported. Model fit was assessed using *R* ^2^ for linear models and McFadden′s pseudo‐*R* ^2^ for logistic regression. Multicollinearity was evaluated using variance inflation factors.

## 3. Results

### 3.1. Participant Flow and Study Population

**Figure 1:**
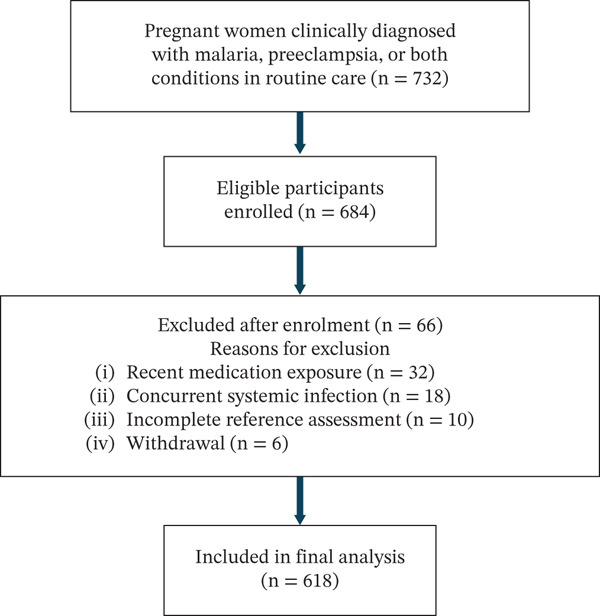
Flow diagram showing screening, enrolment, exclusions and inclusion of participants in the final analysis.

A total of 732 pregnant women who had been clinically diagnosed with malaria, PE or both conditions during routine ANC were screened across participating CHPS compounds, district hospitals and referral facilities during the study period. Of these, 684 participants met the eligibility criteria and were enrolled. After the exclusion of 66 participants based on predefined exclusion criteria, 618 participants were included in the final analysis (Figure 1).

### 3.2. Baseline Sociodemographic and Obstetric Characteristics

A total of 618 pregnant women were included in the analysis and were categorised based on routine facility‐based diagnosis into malaria only (*n* = 304), PE only (*n* = 148), and concurrent malaria and PE (*n* = 166).

Overall, the study population was relatively homogeneous with respect to key sociodemographic and obstetric characteristics. The median maternal age was 30.0 years (IQR 26.0–34.0), and age distribution did not differ significantly across diagnostic categories (*p* = 0.87).

Median gestational age at recruitment was 26.0 weeks (IQR 22.0–30.0). Although women in the malaria‐PE group had a slightly higher median gestational age (27.0 weeks) compared with the malaria‐only and PE‐only groups (both 26.0 weeks), this difference was not statistically significant (*p* = 0.06).

BMI was comparable across all groups, with an overall median of 26.6 kg/m^2^ (IQR 23.5–30.3). No statistically significant differences were observed between diagnostic categories (*p* = 0.88).

ANC utilisation was similarly distributed across groups. The median number of ANC visits was 2.0 (IQR 2.0–3.0) in all diagnostic categories (*p* = 0.71). Parity distribution was also consistent across groups. Overall, 22.7% of participants were nulliparous, and 77.3% were multiparous. The proportion of nulliparous and multiparous women did not differ significantly between diagnostic categories (*p* = 0.47) (Table 1).

**Table 1:** Baseline sociodemographic and obstetric characteristics by routine facility‐based diagnosis.

Variable	Participants (*n* = 618)	Malaria only (*n* = 304)	PE only (*n* = 148)	Malaria and PE (*n* = 166)	*p* value
Age (years) (median, IQR)	30.0 (26.0–34.0)	30.5 (26.8–34.0)	30.0 (25.0–35.0)	30.5 (27.0–34.0)	0.87
Gestational age (median, IQR)	26.0 (22.0–30.0)	26.0 (23.0–29.0)	26.0 (21.0–30.0)	27.0 (24.0–31.0)	0.06
BMI (kg/m^2^) (median, IQR)	26.6 (23.5–30.3)	26.6 (23.7–30.3)	26.1 (23.6–30.5)	26.7 (23.6–30.2	0.88
ANC visits, median (IQR)	2.0 (2.0–3.0)	2.0 (2.0–3.0)	2.0 (2.0–3.0)	2.0 (2.0–3.0)	0.71
Parity, *n* (%)					0.47
Nulliparous	140 (22.7%)	72 (23.7%)	36 (24.3%)	32 (19.3%)	
Multiparous	478 (77.3%)	232 (76.3%)	112 (75.7%)	134 (80.7%)	

Abbreviations: ANC, antenatal care; IQR, interquartile range; PE, pre‐eclampsia.

### 3.3. Routine Facility‐Based Clinical Diagnosis at First Contact

The distribution of routine facility‐based clinical diagnoses varied across levels of care. Most participants were enrolled at CHPS compounds (56.6%), with fewer at district hospitals (26.2%) and referral hospitals (17.2%).

Across all levels of care, malaria‐only diagnosis constituted the largest proportion of routine classifications (49.2%), followed by concurrent malaria and PE (26.9%) and PE‐only diagnosis (23.9%) (Table 2).

**Table 2:** Routine facility‐based clinical diagnosis at the facility level.

Facility level	Malaria only *n* (%)	PE only *n* (%)	Malaria and PE *n* (%)	Total
CHPS compound	162 (26.2%)	85 (13.8%)	103 (16.7%)	350 (56.6%)
District hospital	87 (14.1%)	39 (6.3%)	36 (5.8%)	162 (26.2%)
Referral hospital	55 (8.9%)	24 (3.9%)	27 (4.4%)	106 (17.2%)
Total	304	148	166	618

Abbreviations: CHPS, Community‐based Health Planning and Services; PE, pre‐eclampsia.

### 3.4. Reference Diagnostic Classification

Based on the predefined reference diagnostic criteria, 263 participants (42.6%) were classified as having malaria only, 97 (15.7%) as having PE only and 238 (38.5%) as having concurrent malaria and PE. An additional 20 participants (3.2%) had neither condition (Table 3).

**Table 3:** Reference diagnostic classification.

Reference diagnosis	*n*	%
Malaria only	263	42.6%
PE only	97	15.7%
Malaria and pre‐eclampsia	238	38.5%
Neither malaria nor PE	20	3.2%
Total	618	100%

Abbreviation: PE, pre‐eclampsia.

### 3.5. Diagnostic Concordance Between Routine and Reference Diagnosis

Assessment of agreement between routine facility‐based diagnosis and the reference diagnostic classification demonstrated significant differences in distribution across diagnostic categories (*χ* ^2^(6) = 95.6, *p* < 0.001).

Of the 263 participants classified as malaria only by reference criteria, 164 (62.4%) were concordantly identified as malaria only at routine assessment. Amongst the 97 participants classified as PE only by reference criteria, 43 (44.3%) were similarly classified at routine diagnosis. For the 238 participants classified as having concurrent malaria and PE by reference criteria, 103 (43.3%) were concordantly identified at routine assessment.

Overall, 310 of 618 cases were concordantly classified, corresponding to an observed agreement of 50.2%, with a Cohen′s kappa coefficient of 0.233 (SE = 0.029, *p* < 0.001) (Table 4).

**Table 4:** Diagnostic concordance between routine facility‐based diagnosis and reference diagnostic classification.

Routine diagnosis	Reference diagnosis				
	PE only *n* (%)	PE + M *n* (%)	Malaria only *n* (%)	None *n* (%)	Total (*n*)
PE only	43 (44.3)	33 (13.9)	68 (25.9)	4 (20.0)	148
PE + M	28 (28.9)	103 (43.3)	31 (11.8)	4 (20.0)	166
Malaria only	26 (26.8)	102 (42.9)	164 (62.4)	12 (60.0)	304
Total	97	238	263	20	618

Abbreviations: PE, pre‐eclampsia; PE + M, pre‐eclampsia and malaria.

### 3.6. Diagnostic Performance of Routine Facility‐Based Diagnosis for PE

Routine facility‐based diagnosis demonstrated a sensitivity of 61.8% (95% CI: 56.6–67.0) and a specificity of 62.2% (95% CI: 56.6–67.8) for the detection of PE. The PPV and NPV were 65.9% (95% CI: 60.7–71.1) and 57.9% (95% CI: 52.4–63.4), respectively (Table 5).

**Table 5:** Performance of routine facility‐based diagnosis.

Measure	Estimate (%)	95% confidence interval
Sensitivity	61.8	56.6–67.0
Specificity	62.2	56.6–67.8
Positive predictive value (PPV)	65.9	60.7–71.1
Negative predictive value (NPV)	57.9	52.4–63.4

Abbreviations: NPV, negative predictive value; PPV, positive predictive value.

### 3.7. Clinical Parameters by Reference Diagnostic Classification

There were statistically significant differences in clinical parameters observed across reference diagnostic groups. Median SBP differed across categories (*p* < 0.001), with comparable values in the PE‐only and PE + malaria groups (both 150 mmHg), slightly lower values in the malaria‐only group (147 mmHg) and substantially lower values amongst reference‐negative participants (127 mmHg). DBP similarly varied across groups (*p* < 0.001), with higher median values in the PE‐only (98 mmHg) and PE + malaria groups (97 mmHg).

uPCR also demonstrated significant variation across groups (*p* < 0.001). Median uPCR values were highest in the PE + malaria group (0.341 mg/mg) compared to the other groups. Amongst malaria‐positive groups, parasite density did not differ significantly between women with concurrent PE and malaria (19,250 parasites/*μ*L) and those with malaria only (20,202 parasites/*μ*L) (*p* = 0.13) (Table 6).

**Table 6:** Clinical parameters by reference diagnostic classification.

Variable	PE only (*n* = 97)	PE + malaria (*n* = 238)	Malaria only (*n* = 263)	Reference‐negative (*n* = 20)	*p* value
SBP (mmHg), median (IQR)	150 (144–156)	150 (144–156)	147 (144–152)	127 (123–131)	**< 0.001** ^a^
DBP (mmHg), median (IQR)	98 (95–102)	97 (93–99)	96 (93–98)	84 (82–88)	**< 0.001** ^a^
uPCR (mg/mg), median (IQR)	0.319 (0.211–0.401)	0.341 (0.258–0.442)	0.241 (0.176–0.337)	0.124 (0.106–0.191)	**< 0.001** ^a^
Malaria parasite density (parasites/*μ*L), median (IQR)	—	19,250 (6238–24,084)	20,202 (4507–23,292)	—	**0.13** ^b^

*Note:* Boldened *p* values are significant.

Abbreviations: DBP, diastolic blood pressure; IQR, interquartile range; PE, pre‐eclampsia; SBP, systolic blood pressure; uPCR, urine protein‐to‐creatinine ratio.

^a^Comparison performed across the three disease groups (PE only, PE + malaria and malaria only), excluding reference‐negative cases, using the Kruskal–Wallis test.

^b^Comparison restricted to malaria‐positive groups (PE + malaria and malaria only) using the Mann–Whitney *U* test.

### 3.8. Contribution of Malaria Parasite Density to Diagnostic Discordance

In multivariable linear regression analyses adjusting for maternal age, gestational age, BMI and reference diagnostic classification, log‐transformed parasite density was not significantly associated with SBP (*β* = 1.09, 95% CI: −0.62 to 2.81, *p* = 0.212) or DBP (*β* = −0.24, 95% CI: −1.24 to 0.76, *p* = 0.632). The proportion of variance explained by these models was low (SBP: *R* ^2^ = 0.037; DBP: *R* ^2^ = 0.012).

In contrast, log‐transformed parasite density was independently associated with higher ln(uPCR) levels (*β* = 0.17, 95% CI: 0.08–0.26, *p* < 0.001), with the model explaining 11.8% of the variance (*R* ^2^ = 0.118).

In logistic regression analyses evaluating predictors of diagnostic discordance, log‐transformed parasite density was not independently associated with increased odds of misclassification (aOR 1.09, 95% CI: 0.77–1.55, *p* = 0.631). Neither SBP nor DBP demonstrated a significant association with discordance. Higher ln(uPCR) was not independently associated with diagnostic misclassification (aOR 1.39, 95% CI: 0.98–1.97, *p* = 0.068). The overall explanatory power of the logistic model was limited (McFadden *R* ^2^ = 0.012) (Table 7).

**Table 7:** Multivariable regression models assessing the contribution of malaria parasite density to clinical parameters and diagnostic discordance.

Predictor	SBP (*β*, 95% CI)	DBP (*β*, 95% CI)	ln(uPCR) (*β*, 95% CI)	Misclassification (aOR, 95% CI)
Log10 parasite density	1.09 (−0.62 to 2.81), *p* = 0.212	−0.24 (−1.24 to 0.76), *p* = 0.632	0.17 (0.08–0.26), ***p* <0.001**	1.09 (0.77–1.55), *p* = 0.631
Age (years)	0.06 (−0.08 to 0.21), *p* = 0.389	0.09 (−0.001 to 0.17), *p* = 0.054	−0.004 (−0.011 to 0.003), *p* = 0.283	0.995 (0.97–1.03), *p* = 0.765
Gestational age (weeks)	−0.06 (−0.22 to 0.10), *p* = 0.450	−0.05 (−0.15 to 0.04), *p* = 0.273	−0.002 (−0.010 to 0.006), *p* = 0.653	0.985 (0.95–1.02), *p* = 0.369
BMI (kg/m^2^)	−0.03 (−0.07 to 0.02), *p* = 0.204	−0.01 (−0.04 to 0.01), *p* = 0.353	0.0002 (−0.002 to 0.002), *p* = 0.841	1.01 (0.99–1.04), *p* = 0.256
ln(uPCR)	—	—	—	1.39 (0.98–1.97), *p* = 0.068
Model fit	*R* ^2^ = 0.037	*R* ^2^ = 0.012	*R* ^2^ = 0.118	McFadden *R* ^2^ = 0.012

*Note:* *β* coefficients represent the adjusted change in outcome per unit increase in the predictor. Odds ratios (aORs) represent adjusted odds of diagnostic discordance. Parasite density was log10‐transformed. uPCR was natural log‐transformed. Boldened *p* value is significant.

Abbreviations: DBP, diastolic blood pressure; SBP, systolic blood pressure; uPCR, urine protein‐to‐creatinine ratio.

## 4. Discussion

This study evaluated diagnostic concordance and misclassification amongst pregnant women clinically diagnosed with malaria, PE or both conditions across multiple tiers of care in a malaria‐endemic setting. The inclusion of participants from CHPS compounds, district hospitals and referral facilities reflects real‐world diagnostic practice across primary, secondary and tertiary care settings in this population.

The study population was broadly comparable across routine diagnostic categories with respect to maternal age, gestational age, BMI, ANC utilisation and parity. The absence of statistically significant baseline differences across groups suggests that the diagnostic groups were not structurally different by sociodemographic or obstetric characteristics. This comparability strengthens the internal validity of the analyses of this study, as differences in clinical parameters or diagnostic performance are less likely to be attributable to demographic imbalance and more likely to reflect diagnostic processes or disease‐related factors.

The predominance of malaria‐only diagnoses at routine assessment is consistent with the epidemiological profile of malaria‐endemic regions [10]. Malaria remains a major contributor to maternal morbidity in sub‐Saharan Africa [11, 12], and its frequent coexistence with hypertensive disorders complicates clinical differentiation [13].

Reference classification identified a higher proportion of concurrent malaria and PE cases than routine facility‐based diagnosis. This discrepancy suggests potential underdetection of concurrent pathology during initial clinical assessment. In many resource‐limited settings, routine diagnosis of PE relies on symptom assessment, blood pressure measurement and qualitative proteinuria testing using dipstick methods [14, 15]. Although widely implemented, dipstick proteinuria has well‐documented limitations in sensitivity and specificity [16, 17], potentially contributing to both over‐ and underdiagnosis of PE. Furthermore, malaria has been associated with renal involvement, endothelial activation and transient proteinuria [18, 19], thereby creating a biological overlap with core diagnostic features of PE. Such overlap may predispose clinicians either to attribute proteinuria and hypertension to malaria alone or to misclassify malaria‐related renal changes as PE [20].

Facility‐level variation further contextualises these findings. Differences in diagnostic classification across tiers of care likely reflect variability in training, diagnostic resources and clinical experience. Primary‐level facilities such as CHPS compounds frequently operate with limited laboratory capacity and may rely heavily on syndromic evaluation [21], whereas referral centres are more likely to have access to quantitative laboratory testing and specialist review. This is consistent with evidence indicating that diagnostic accuracy for hypertensive disorders of pregnancy can vary substantially by facility level [22, 23]. Moreover, in malaria‐endemic environments, the clinical suspicion of malaria may influence the interpretation of overlapping signs and laboratory findings [24–26] such that healthcare facilities without the requisite specialists′ review may likely misclassify PE as malaria.

Diagnostic concordance between routine assessment and reference classification was modest, with only half of the cases correctly classified. Similar levels of diagnostic concordance for hypertensive disorders of pregnancy have been reported in other low‐resource settings [27, 28], where reliance on clinical assessment and limited laboratory support constrains diagnostic precision [29]. Concordance was highest for malaria‐only diagnoses and lower for PE only and concurrent malaria‐PE. This suggests that PE, particularly when coexisting with malaria, may be misclassified in this population [30].

From a clinical perspective, such misclassification has implications for the local population. Underdiagnosis of PE may delay timely initiation of antihypertensive therapy, magnesium sulphate prophylaxis and appropriate referral, thereby increasing the risk of maternal and perinatal complications. Conversely, overdiagnosis may result in unnecessary hospitalisation, premature delivery or unwarranted pharmacological intervention in settings where healthcare resources are already constrained.

The diagnostic performance of routine assessment for PE further highlights these challenges. With sensitivity and specificity both around 62%, a substantial proportion of true PE cases were not identified, potentially delaying appropriate management or timely referral. Given that PE is a leading cause of maternal mortality globally and in sub‐Saharan Africa [31], missed diagnoses at first contact may translate into preventable adverse outcomes for mothers and infants.

These findings highlight the need for strengthened screening protocols, improved standardisation of blood pressure measurement and more reliable assessment of proteinuria to enhance diagnostic precision and reduce avoidable misclassification in routine ANC.

After adjusting for maternal and clinical covariates, malaria parasite density was not independently associated with SBP or DBP. This suggests that malaria parasite burden may not contribute meaningfully to blood pressure elevation in this cohort. In contrast, malaria parasite density was independently associated with a higher uPCR, supporting the hypothesis that malaria contributes to renal protein leakage independent of blood pressure [32, 33].

Malaria parasite density was not independently associated with diagnostic discordance. This finding suggests that malaria infection may not be the principal driver of misclassification in this population. Furthermore, neither blood pressure nor uPCR independently predicted discordance, suggesting that misclassification may arise less from physiological overlap and more from contextual diagnostic factors.

In particular, the reliance on qualitative dipstick proteinuria testing, which is subject to variability due to urine concentration and observer interpretation, likely contributes to inconsistent classification of PE. Similarly, variability in blood pressure measurement, including differences in technique, device accuracy and adherence to standardised protocols at primary‐level facilities, may further compound diagnostic inaccuracy.

Previous evaluations of hypertensive disorder diagnosis in low‐resource settings have similarly identified workflow limitations and measurement variability as primary contributors to diagnostic inaccuracy [34, 35]. The findings of this study therefore reinforce the need for facility‐level improvements, including standardisation of blood pressure measurement, training of frontline healthcare providers and access to quantitative proteinuria assessment methods. Addressing these gaps is essential to reducing diagnostic discordance and improving early identification of PE in malaria‐endemic settings.

The findings of this study should be interpreted within the context of some limitations. Malaria parasite density was quantified using peripheral blood samples, which may not accurately reflect placental sequestration or tissue‐level parasitic burden. As a result, peripheral parasitaemia may not represent the true parasite load at the placental level, where the primary pathological processes occur. This discrepancy implies that the local inflammatory and endothelial perturbations within the placenta could differ from what is estimated based on peripheral parasite density alone. Additionally, the study was conducted within a defined malaria‐endemic setting, and the results may not be generalisable to populations with different patterns of malaria transmission or healthcare infrastructure.

Despite these limitations, the study provides clinically meaningful insights into the diagnostic challenges arising from the coexistence of malaria and PE and their implications for maternal care in resource‐constrained settings. By examining real‐world diagnostic processes across multiple tiers of care, the findings may be directly applicable to routine antenatal practice in low‐resource, malaria‐endemic environments with similar demographics and healthcare structure. Furthermore, the application of predefined reference diagnostic criteria enhances the robustness of diagnostic comparison and strengthens the interpretability of observed concordance and misclassification patterns.

## 5. Conclusion

This study demonstrates that routine diagnosis of PE in malaria‐endemic settings is characterised by moderate sensitivity and specificity, with substantial misclassification, especially when malaria and PE coexist. The findings highlight the need to strengthen antenatal diagnostic capacity at primary‐level facilities. Policy efforts should focus on standardising blood pressure measurement, improving access to reliable quantitative proteinuria assessment and implementing clinical protocols that explicitly recognise the potential overlap between malaria and hypertensive disorders in pregnancy. These will improve diagnostic precision at first contact and improve maternal outcomes in resource‐constrained, malaria‐endemic environments.

## Funding

No funding was received for this research. The research was performed as part of the employment of the University of Worcester. University of Worcester was not involved in the manuscript writing, editing, approval, or decision to publish.

## Conflicts of Interest

The authors declare no conflicts of interest.

## Data Availability

The data that support the findings of this study are available from the corresponding author upon reasonable request.
